# Advances in MRI-Based Assessment of Rectal Cancer Post-Neoadjuvant Therapy: A Comprehensive Review

**DOI:** 10.3390/jcm13010172

**Published:** 2023-12-28

**Authors:** Joao Miranda, Pamela Causa Andrieu, Josip Nincevic, Lucas de Padua Gomes de Farias, Hala Khasawneh, Yuki Arita, Nir Stanietzky, Maria Clara Fernandes, Tiago Biachi De Castria, Natally Horvat

**Affiliations:** 1 Department of Radiology, Memorial Sloan Kettering Cancer Center, 1275 York Avenue, New York, NY 10065, USA; nincevij@mskcc.org (J.N.); yukiarita1113@gmail.com (Y.A.); fernanm3@mskcc.org (M.C.F.);; 2 Department of Radiology, University of Sao Paulo, R. Dr. Ovidio Pires de Campos, 75 Cerqueira Cesar, Sao Paulo 05403-010, Brazil; 3 Department of Radiology, Mayo Clinic, 200 First St. SW, Rochester, MN 55905, USA; causaandrieu.pamela@mayo.edu; 4 Department of Radiology, Hospital Sirio-Libanes, Rua Dona Adma Jafet, 91—Bela Vista, Sao Paulo 01308-050, Brazil; lucasdpadua@gmail.com; 5 Department of Radiology, Allianca Saude, Av. Pres. Juscelino Kubitschek, 1830, Sao Paulo 01308-050, Brazil; 6 Department of Radiology, University of Texas Southwestern, 5323 Harry Hines Blvd, Dallas, TX 75390, USA; hala.khasawneh@utsouthwestern.edu; 7 Department of Radiology, Keio University School of Medicine, 35 Shinanomachi, Shinjuku-ku, Tokyo 160-8582, Japan; 8 Division of Diagnostic Imaging, The University of Texas MD Anderson Cancer Center, Houston, TX 77030, USA; nstanietzky@mdanderson.org; 9 Department of Gastrointestinal Oncology, Moffit Cancer Center, 12902 USF Magnolia Drive, Tampa, FL 33612, USA; tiago.biachi@moffitt.org; 10 Morsani College of Medicine, University of South Florida, 4202 E. Fowler Avenue, Tampa, FL 33620, USA

**Keywords:** rectal cancer, neoadjuvant therapy, magnetic resonance imaging, treatment response

## Abstract

Rectal cancer presents significant diagnostic and therapeutic challenges, with neoadjuvant therapy playing a pivotal role in improving resectability and patient outcomes. MRI serves as a critical tool in assessing treatment response. However, differentiating viable tumor tissue from therapy-induced changes on MRI remains a complex task. In this comprehensive review, we explore treatment options for rectal cancer based on resectability status, focusing on the role of MRI in guiding therapeutic decisions. We delve into the nuances of MRI-based evaluation of treatment response following neoadjuvant therapy, paying particular attention to emerging techniques like radiomics. Drawing from our insights based on the literature, we provide essential recommendations for post-neoadjuvant therapy management of rectal cancer, all within the context of MRI-based findings.

## 1. Introduction

Colorectal cancer remains a significant health challenge; it is the second most common cause of cancer mortality in the United States in 2023. Furthermore, despite a decline in the overall incidence rates of colorectal cancer, the proportions of younger patients and patients with rectal cancer among newly diagnosed cases of colorectal cancer have notably increased over recent years. This underscores the urgency for effective treatment strategies and assessment methodologies for the evolving demographic of patients with colorectal cancer [1].

Recent years have seen a shift from traditional surgery towards organ-preserving treatments, including non-operative management (NOM) [2,3,4,5] for patients with rectal cancer who achieve pathological complete response after neoadjuvant therapy [5,6,7]. However, while such novel organ-preserving strategies offer numerous advantages over traditional surgery, there is a crucial knowledge gap in the treatment response assessment pertaining to these strategies. Of several modern imaging techniques, rectal magnetic resonance imaging (MRI) is promising for the purpose of treatment response assessment, but to date, the literature remains lacking concerning its use in the post-neoadjuvant therapy setting in patients with rectal cancer [4,8,9,10,11,12,13].

Here, we provide a comprehensive, up-to-date review of the advances in MRI-based assessment of rectal cancer in the post-neoadjuvant therapy setting. In response to the critical need for a refined treatment response assessment method in this setting, we also provide an updated, systematic approach for treatment response assessment in the post-neoadjuvant therapy setting using rectal MRI, incorporating the latest results and terminology from the literature. This will not only aid in the immediate treatment planning but also have broader implications for determining the eligibility criteria for NOM and other organ preservation strategies.

## 2. Overview of Neoadjuvant Therapy

### 2.1. Neoadjuvant Therapy

Neoadjuvant therapy refers to any treatment administered before the standard definitive surgical resection (i.e., total mesorectal excision) and is usually indicated in patients with locally advanced rectal cancer [14,15]. While the National Comprehensive Cancer Care guidelines recommend neoadjuvant therapy to any patient with T3-, T4-, T1-2 with N+, or locally unresectable or inoperable cancer due to coexisting medical conditions [15], the European Society for Medical Oncology emphasizes the use of MRI to stratify patients into different treatment subgroups [14]. Remarkably, neoadjuvant therapy allows patients who achieve a complete response to enter a “watch-and-wait” protocol, also known as NOM, to avoid surgical complications [16]. Extensive research has been conducted to propose different neoadjuvant therapy strategies to improve patient outcomes. In the following paragraphs, we provide insight into these strategies.

The traditional neoadjuvant therapy approach is the administration of capecitabine or infusion fluorouracil chemotherapy with long- or short-course radiotherapy (RT) [15]. While long-course RT delivers 45–50 Gy over 25–30 fractions, short-course RT provides 25 Gy over five fractions (commonly described as “5 × 5”) in just a week [15]. Short-course RT has been shown to lead to a reduced recurrence rate but is associated with increased local side effects [17,18,19].

*Total neoadjuvant therapy* (TNT) is an approach that delivers both systemic chemotherapy and chemoradiotherapy (CRT) before surgery, consisting of induction chemotherapy followed by CRT or consolidation chemotherapy administered after CRT [15,20]. The goals are to decrease the rate of distant metastasis and to increase the rate of clinical complete response (up to 40% in a recent meta-analysis) [21]. Some of the relevant clinical trials on TNT include the following:

- The *Organ Preservation of Rectal Adenocarcinoma (OPRA)* trial compared patients on INCT-CRT (Induction Chemotherapy with Chemoradiotherapy) and patients on CRT-CNCT (Chemoradiotherapy with Consolidation Chemotherapy). Remarkably, approximately 75% of both patient groups underwent the NOM protocol and both patient groups had similar outcomes in terms of 3-year disease-free survival (76% and 76%, log-rank *p* = 0.98), local recurrence-free survival (94% and 94%, log-rank *p* = 0.78), distant metastasis-free survival (84% and 82%, log-rank *p* = 0.67), and local tumor regrowth (40% and 27%, log-rank *p* = 0.03). In terms of rectum preservation, however, more patients from the CRT-CNCT group achieved rectum preservation compared to patients from the INCRT-CRT group (60% [95% confidence interval [CI]: 52–68] vs. 47% [95% CI: 39–56]), which justifies initially providing INCT-CRT in cases where NOM is preferred [20].
- The *Rectal cancer And Pre-operative Induction Therapy Followed by Dedicated Operation (RAPIDO)* trial showed a decreased rate of distant metastasis at 3 years of follow-up, reflected by the rate of disease-related treatment failure, in patients treated with experimental short-course RT, TNT, and total mesorectal excision (rate of ~24%) compared to patients treated with standard long-course CRT, total mesorectal excision, and optional adjuvant chemotherapy (rate of ~30%), albeit both patient groups had comparable rates of locoregional failure [22]. The recently published 5-year follow-up results showed a similar pattern in terms of distant metastasis; however, the rate of locoregional failure, reflected by the rate of locoregional recurrence, was higher in patients treated with the experimental approach (rate of ~12%) compared to patients treated with standard approach (rate of ~8%). These results highlight the necessity of further refining the neoadjuvant therapy approach [23].
- The *Unicancer Gastrointestinal Group and Pertenariat de Recherche en Oncologie Digestive (PRODIGE 23)* trial compared one group of patients who received standard CRT, total mesorectal excision, and adjuvant FOLFOX (“standard-of-care”) and another group of patients who received neoadjuvant FOLFIRINOX therapy (“TNT”), CRT (radiotherapy and fluorouracil), TME, and adjuvant FOLFOX or capecitabine. The TNT group showed increased 3-year disease-free survival (76% vs. 69%; hazard ratio (HR) 0.69 [95% CI: 0.49−0.97]; *p* = 0.034), increased 3-year rate of metastasis-free survival (79% vs. 72%; HR 0.64, [95% CI: 0.44–0.93] (*p* = 0.017), and increased pathologic complete response rate (12% vs. 28%) (*p*  <  .001) [24]. The 7-year follow-up presented in the last American Society of Clinical Oncology meeting showed that the TNT group had an absolute increase of 7.6% for disease-free survival, 6.9% for overall survival, 9.9% for metastasis-free survival, and 5.7% for cancer-specific survival, as well as decreased locoregional relapse (5.3% vs. 8.1%, *p* = 0.38) [25].
- Recently published results from the *Chemotherapy Alone or Chemotherapy Plus Radiation Therapy in Treating Patients with Locally Advanced Rectal Cancer Undergoing Surgery (PROSPECT)* trial aimed to evaluate the outcomes of patients who received neoadjuvant chemotherapy but without RT among patients with T2 node-positive, T3 node-negative, or T3 node-positive and candidates for sphincter-sparing surgery. They found that neoadjuvant chemotherapy (FOLFOX) was non-inferior to the standard CRT approach in regard to disease-free survival (HR 0.92 [90.2% CI: 0.74 to 1.14]) (*p* = 0.005) [26].

Finally, a novel approach with immunotherapy has been proposed for a subgroup of patients with locally advanced rectal cancer and mismatch repair deficiency: Cercek et al. conducted a prospective phase 2 study involving 12 patients who completed treatment with dostarlimab (anti-PD-1 monoclonal antibody), and all of them had a complete clinical response and underwent NOM at the time of publication of the phase 2 results [27]. Other more recent results from other investigators have supported the results from Cercek et al.’s phase 2 study [28,29].

### 2.2. Response Assessment

Recently, an international consensus was formulated to standardize the treatment response categorization of patients with rectal cancer who are undergoing organ preservation strategies after CRT [30]. The core objective of this consensus is to offer researchers and clinicians a well-defined, standardized framework for assessing and conveying the efficacy of organ preservation strategies. According to this consensus, following treatment with an organ preservation strategy, patients are categorized into three specific groups based on findings from digital rectal examination, endoscopy, and MRI, as follows:

- Clinical complete response: This response is reflected by normal findings on digital rectal examination, an unremarkable rectal wall with or without fibrosis, and no adenopathy on MRI. Endoscopy findings are not required for clinical complete response categorization.
- Near-complete response: This response is reflected by smooth indurations and/or minimal mucosal abnormalities on digital rectal examination; irregular or smooth mucosa irregularities, superficial ulcer, and persistent erythema on endoscopy; and apparent decrease in size with predominant fibrosis and without or with borderline lymph nodes on MRI.
- Incomplete response: This response is reflected by a palpable tumor on digital rectal examination as well as a visible tumor with or without nodal regression on endoscopy and MRI.

## 3. Restaging MRI Protocol

### 3.1. Preparation

Spasmolytic agents are usually part of the motion-mitigation strategy within the restaging MRI protocol, but their use is not mandatory. The survey led by Gollub et al. found that 47% of academic institutions use spasmolytics (glucagon, 1 mg, administered intravenously/intramuscularly/subcutaneously; or hyoscyamine butyl bromide, 20 mg, administered intravenously) for rectal cancer MRI in the baseline or neoadjuvant setting [31]. Spasmolytic agents reduce peristalsis-causing artifacts, especially on the diffusion-weighted imaging (DWI) sequence of the upper rectum. Fasting for 2 h before scanning decreases small bowel peristalsis [32].

Of note, while the baseline MRI protocol does not require a microenema, it is crucial to use a microenema in the restaging MRI protocol to minimize gas-related artifacts, consequently enhancing the quality of DWI. It is recommended to administer a microenema 15 min before the scan [33,34].

Numerous guidelines suggest a minimum field strength of 1.5 Tesla (T) for MRI in rectal cancer cases. The choice between 1.5 T and 3.0 T for rectal cancer imaging lacks consensus. While MRI at 3 T offers several advantages, including faster image acquisition and improved spatial resolution with a higher signal-to-noise ratio, these advantages come at the expense of higher susceptibility artifacts, which might impact DWI particularly [32,35].

### 3.2. Coils

The patient should be positioned in a supine posture. Instead of endorectal coils, which can lead to discomfort and rectal distention while potentially affecting the measured distance between the tumor and circumferential resection margin [36], pelvic phased-array surface coils are recommended. These surface coils should be positioned with their lower edge just below the pubic bone or, for cases involving low rectal tumors, placed at least 10 cm below the symphysis pubis. The upper edge of the coil should align with the sacral promontory [32].

### 3.3. Sequences

*Large field-of-view (FOV) T2-weighted imaging* is essential for evaluating tumor extension beyond the mesorectal compartment, especially when assessing nodes outside the total mesorectal excision zone. These images should encompass the region from the origin of the superior mesenteric artery to the inguinal regions [32,37].

*High-resolution T2-weighted imaging* should have a field of view (FOV) of 16–20 cm, slice thickness of 2–3 mm, in-plane resolution less than 1 × 1 mm, with no gap between slices, and an echo time (TE) of 80–110 ms, depending on the field strength. High-resolution, small FOV T2-weighted imaging, in particular, is crucial for assessing delicate structures that demand maximum contrast resolution, such as the rectal wall, mesorectal fat, mesorectal lymph nodes, and peritoneal reflection. This sequence is vital not only for T and N staging but also for detailed scrutiny of extramural vascular invasion (EMVI), differentiation between mesorectal tumor deposits and lymph nodes, and the evaluation of peritoneal reflection involvement [38]. The sequence should involve imaging in the oblique axial, oblique coronal, and true sagittal planes relative to the tumor’s location; this approach is critical to prevent volume averaging and to avoid misinterpretation of tumor extension [39]. In cases of extensive tumors, acquiring oblique axial and oblique coronal T2-weighted images at different angles is sometimes necessary to assess orientation and extension at various levels. Oblique small FOV T2-weighted imaging aligned with the anal canal assists in the local staging of low rectal tumors and their relationship to the sphincter complex.

*DWI* is a functional imaging sequence that relies on rate differences in the random motion of the water molecules within the tissue of interest. A higher signal indicates the restricted ability of water molecules to diffuse within the microenvironment. Restricted diffusion is associated with abscesses, hypercellular tumors, and benign and malignant lymphatics (lymph nodes, spleen). DWI adds to the accuracy of conventional morphological MRI sequences, especially in the post-treatment setting. Even though DWI cannot distinguish benign and malignant lymph nodes, it is a helpful tool in detecting and differentiating such lymph nodes from common benign mimickers like varicose veins and phleboliths, among others [40]. The use of multiple b values (50 s/mm^2^, 400 s/mm^2^, and 800 s/mm^2^) in a single DWI acquisition not only saves time but also enhances diagnostic capabilities [32].

*Small FOV DWI* is essential in evaluating residual tumors to determine a clinical complete response and thereby determine further selection for NOM [41]. It is important to emphasize that DWI can detect recurrent tumors before they become apparent on endoscopy [42]. Small FOV DWI has been shown to provide better subjective image quality and higher accuracy for post-treatment reevaluation compared to full FOV DWI [43,44]. Most protocols use b values ≥ 1000 s/mm^2^ to minimize false T2 shine-through from submucosal and luminal edema. One study compared the use of two high b values (b = 1000 s/mm^2^ and b = 2000 s/mm^2^), showing that b = 2000 s/mm^2^ resulted in higher tumor conspicuity compared to b = 1000 s/mm^2^ [45]. With small FOV DWI, luminal gas has been shown to be the main cause of susceptibility artifacts, potentially obscuring actual lesions or creating pseudo lesions [32].

### 3.4. Intravenous Contrast

Even though the reliability of the postcontrast sequences in the posttreatment setting is not as accurate, for example, because of the enhancement in the posttreatment and inflammatory changes, both the Society of Abdominal Radiology and the European Society of Gastrointestinal and Abdominal Radiology suggest that intravenous contrast may be considered as an optional tool in specific scenarios, for example in situations where image quality is compromised by artifacts [31,46].

## 4. Rectal MRI Response Assessment

### 4.1. Why Assessment Matters

Conducting an MRI after neoadjuvant CRT is crucial for assessing the cancer’s response to treatment, detecting new disease sites, re-evaluating the extent of the disease, and planning further treatment (e.g., surgery or NOM).

### 4.2. When to Evaluate

The optimal timing for the initial assessment of tumor response remains a point of contention within the field. Research suggests that the rate of achieving pathological complete response increases significantly after 12 weeks following radiotherapy [47]. Nonetheless, some surgical groups express reservations about performing operations beyond 8 weeks following RT. Their concerns are rooted in fears of radiation-induced pelvic fibrosis and associated surgical complications [48]. These apprehensions underscore the importance of early identification of poor or incomplete responders.

Efforts to shift the decision point from the conventional 6- to 8-week post-radiotherapy period to a later window of 10 to 12 weeks may not necessarily adversely impact surgery-related morbidity or mortality [49]. Moreover, extending the surveillance window might prove advantageous since prolonged intervals may be useful to enhance response rates [50]. It also offers the opportunity to initiate consolidation CRT for metastatic high-risk patients, a decision that could potentially benefit from TNT [49]. This strategic approach emphasizes the need for a more comprehensive understanding of when to assess tumor response and its potential implications for patient management.

### 4.3. Pre-Assessment Preparations

Before presenting findings from restaging MRI of the rectum, it is crucial to delve into the patient’s clinical history, including the results of digital rectal examinations and any conducted endoscopic procedures. It is equally important to consider the type of treatment administered, whether it is CRT or TNT, along with the time elapsed since the completion of the last treatment session [51]. When the availability of prior clinical records is limited or absent, drawing substantiated conclusions becomes an intricate challenge.

Moreover, conducting an assessment of the baseline MRI scan, when available, assumes great significance as a preparatory step before embarking on the interpretation of the restaging rectal MRI. This initial examination serves to illuminate critical facets, encompassing tumor localization, tumor characteristics, and the potential presence of mucinous components. Notably, following neoadjuvant therapy, it is essential to recognize that the normal rectal wall near or opposite the treated tumor may experience edematous thickening, which could be mistakenly identified as a residual tumor, often termed “pseudotumor,” by some observers [52]. Thus, establishing a correlation with the baseline rectal MRI stands as a highly beneficial practice, aiding in the precise determination of the tumor bed’s location [53]. Scar tissue should follow the same distribution and shape as the primary tumor [54]. This integrated approach enhances the accuracy of interpretation and minimizes the potential for misinterpretations.

### 4.4. How to Evaluate the Tumor Response

In managing locally advanced rectal cancer, radiologists are pivotal in assessing treatment response, specifically during the “tumor assessment” phase. Successful response to treatment in rectal tumors typically results in morphologic changes on imaging, including size reduction and fibrotic transformation.

#### 4.4.1. Size-Reduction-Based Assessment

Although the literature suggests that a 60–80% reduction in tumor volume is associated with positive treatment response [55], Response Evaluation Criteria in Solid Tumors (RECIST) criteria are not commonly used in rectal cancer due to challenges in measuring irregularly shaped tumors.

#### 4.4.2. Fibrotic Transformation-Based Assessment

Untreated non-mucinous tumors exhibit intermediate signal intensity on T2-weighted MRI, which is lower than that of fat tissue but higher than that of the normal muscular bowel wall layer. With fibrosis, the signal drops significantly, rendering the tumor bed markedly hypointense.

Some centers utilize the MRI tumor regression grade (mrTRG) scale, a modified version of the TRG scale used in pathology [56]. mrTRG employs a simple 1–5 scoring system based on the degree of remaining tumor and amount of fibrosis after neoadjuvant therapy:

- mrTRG 1—no or minimal fibrosis visible (thin linear scar) with low signal intensity on T2WI and no tumor signal (intermediate signal intensity);
- mrTRG 2—dense fibrosis and no tumor signal;
- mrTRG 3—predominantly fibrotic and obvious measurable areas of tumor signal;
- mrTRG 4—predominantly tumor signal with minimal fibrosis;
- mrTRG 5—only tumor or increased tumor since baseline.

This scoring system is associated with disease-free and overall survival [13] and has modest accuracy for the detection of pathologic complete response [57]. However, it is important to note that there is a limited correlation between mrTRG and pathologic TRG. A meta-analysis by Jang et al. found that ymrTRG scores of 1–2, which correspond to complete or good radiological regression, had a sensitivity of 70–71% and a specificity of 62–68% [58,59]. Moreover, the inter-reader agreement of ymrTRG is highly variable, with kappa values ranging from 0.25 to 0.8 [60,61,62].

#### 4.4.3. Updates on Treatment Assessment

**Figure 1:**
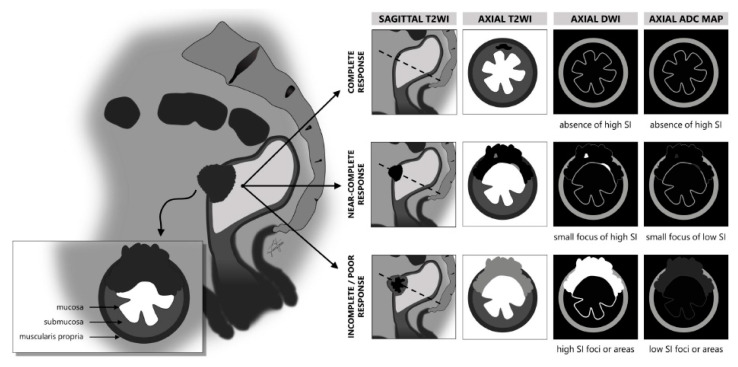
MRI-based morphologic response categories in rectal cancer. This figure delineates the newly introduced terminology by the Society of Abdominal Radiology Colorectal and Anal Cancer Disease-Focused Panel for classifying morphologic responses in colorectal and anal cancers using MRI. The visual guide showcases the following: Complete Response: characterized by the remarkable vanishing of intermediate signal intensity on T2-weighted imaging (T2WI), suggestive of substantial tumor size reduction, often seen as a scar in the mucosal or submucosal layers or normalized rectal wall structure; Near-Complete Response: indicative of significant tumor regression, near-complete response is a state that may evolve into a complete response with time. This stage may exhibit minimal but identifiable diffusion restriction; Incomplete Response: marked by a decrease in tumor volume with a residual detectable tumor on imaging, signifying persistent diffusion restriction and intermediate T2 signal within the tumor bed, necessitating further treatment consideration. Each category is supported by corresponding MRI findings (T2WI, diffusion-weighted imaging (DWI), and DWI-derived apparent diffusion coefficient (ADC) map findings) to aid in the comprehensive evaluation and accurate classification of treatment response.

New terminology was introduced in 2023 by the Society of Abdominal Radiology Colorectal and Anal Cancer Disease-Focused Pane (SAR DFP) [63] to facilitate precise and comprehensible morphologic response characterization. According to this new terminology, treatment response at restaging is categorized into complete response (CR), near-complete response (nCR), or incomplete response (iCR) (Figure 1). In this section, we delve into the treatment assessment process, unraveling the intricacies of tumor-related changes on T2-weighted imaging and DWI while elucidating each type of response.

**Figure 2:**
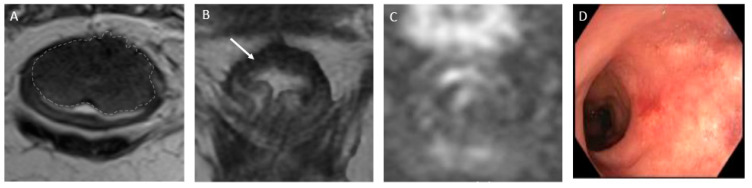
Complete response after neoadjuvant chemotherapy in a 54-year-old man with low rectal adenocarcinoma. (**A**) Baseline axial T2-weighted MR image shows a low rectal tumor (dotted line). (**B**) Axial T2-weighted MR image after the completion of neoadjuvant chemoradiotherapy shows a thick hypointense scar at the site of the treated tumor (arrow). No diffusion restriction was present on diffusion-weighted images (**C**), and no residual malignancy was identified at endoscopy (**D**). The patient was offered a watch-and-wait (non-operative management) strategy and has been without evidence of disease for 36 months.

**Figure 3:**
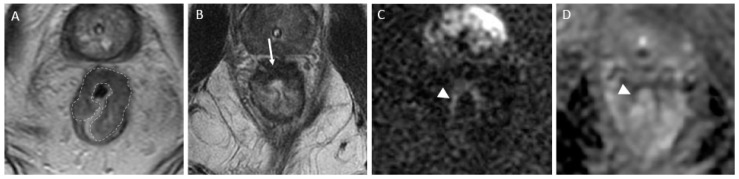
Near-complete response in a 65-year-old man with middle rectal adenocarcinoma. (**A**) Baseline axial T2-weighted MR image shows an intermediate-signal-intensity, near-circumferential low rectal tumor (dotted line). (**B**) Axial T2-weighted MR image after the completion of neoadjuvant chemoradiotherapy shows a slight response with a small amount of fibrosis and residual tumor signal intensity (arrow). Axial diffusion-weighted image (**C**) shows high signal intensity (arrowhead), and the axial apparent diffusion coefficient map (**D**) shows a corresponding low signal intensity (arrowhead), in keeping with restricted diffusion at the nonfibrotic portion of the tumor.

**Figure 4:**
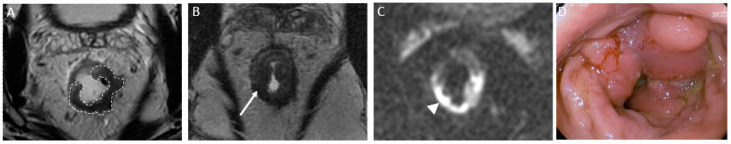
Incomplete response in a 60-year-old man with low/middle rectal adenocarcinoma. (**A**) Baseline axial T2-weighted MR image shows an intermediate-signal-intensity, near-circumferential low rectal tumor (dotted line). (**B**) Axial T2-weighted MR image after the completion of neoadjuvant chemoradiotherapy shows persistent intermediate signal intensity (arrow) representing incomplete response and residual tumor. (**C**) Axial diffusion-weighted image shows high signal intensity (arrowhead). (**D**) The residual malignancy was identified at endoscopy. The patient underwent total mesorectal excision.

(a) *CR* signifies the remarkable disappearance of T2 intermediate signal, indicating a significant reduction in tumor size and suggesting a highly favorable response to treatment. Changes in T2-weighted imaging and DWI pertaining to CR are described below and exemplified in Figure 2:
  - *T2-weighted imaging*—In T2-weighted imaging, CR can be represented as a linear or crescent-shaped scar within the mucosal/ submucosal layers or even the normalization of the rectal wall. It is known that rectal wall normalization can be seen in 5% of cases and is suggestive of CR [64].
  - *DWI*—CR on DWI is characterized by the absence of high signal intensity on high b-value DW images [65,66,67,68]. It is essential to compare DW images at restaging with baseline images and with the normal rectum as references. This can be especially valuable in identifying CR in small, subcircumferential scars [69].
(b) *nCR* serves as a transitional state between CR and other responses, with substantial regression evident. Of note, the term nCR emerged only recently, driven by the observation that a significant proportion of patients who display very good yet incomplete responses during the first assessment do ultimately achieve a CR when provided with a longer interval before re-assessment (26) (Figure 3). nCR retains a trace of diffusion restriction post-neoadjuvant therapy, underscoring ongoing positive changes. In cases where tumor signal or diffusion restriction persists after one or two short-term follow-up evaluations, the case should be reclassified as iCR and considered unsuitable for observation.
(c) *iCR* characterizes the scenario where tumor volume experiences a reduction, but discernible residual tumor persists. This response type manifests through persistent diffusion restriction and the persistence of T2 intermediate signal within the tumor bed (Figure 4).

It is important to note that the new terminology grouped CR and nCR patients together, as they can both benefit from vigilant monitoring, which may lead to improved treatment outcomes [58,70]. The majority of cases classified as CR and nCR (ranging from 73 to 99%) typically transition to CR during short-term follow-up evaluations conducted within 6–12 weeks after neoadjuvant treatment [71].

Conversely, patients exhibiting iCR or no response on MRI are not ideal candidates for NOM. These indicators are linked with suboptimal responses, potentially necessitating alternative therapeutic strategies.

#### 4.4.4. Mucinous Rectal Cancer

The assessment of mucinous rectal cancer post-neoadjuvant therapy poses unique imaging challenges. There have been significant advances in the treatment of such patients that have greatly benefited the overall survival rate, particularly in the area of neoadjuvant immunotherapy [72]. A decrease in tumor bulk is readily identifiable, but many of these cancers that respond to therapy also leave residual areas of mucin. MRI is limited in its ability to distinguish between cellular and acellular mucinous components, which is a crucial distinction, particularly when considering an NOM approach [73,74].

Multiple mrTRG systems have been proposed for this purpose. One such grading system was developed by Park et al. [75] in 2017. This consists of an MRI staging of TRG 1–5 attempting to emulate the pathologic Mandard TRG stage. The common theme of the many mrTRG systems is that the likelihood of residual viable tumor cells is inversely proportional to the amount of intermediate T2 signal of the remaining soft tissue and/or fibrosis.

**Figure 5:**
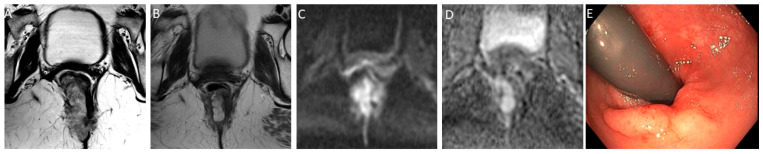
A 57-year-old female diagnosed with mucinous rectal carcinoma. Initial T2-weighted oblique axial image reveals a large low mucinous rectal carcinoma invading the external sphincter (**A**). Following total neoadjuvant therapy, a considerable decrease in tumor size was observed about four months later, with some mucin deposits persisting (**B**). While the intermediate T2 signal has diminished, indicative of a response to treatment, slender strands of fibrosis remain. b-800 diffusion-weighted imaging (**C**) and apparent diffusion coefficient (ADC) mapping (**D**) are somewhat compromised in their diagnostic utility by the ‘T2 shine-through” effect associated with mucin. Furthermore, endoscopic examination identified a healed ulcer with overlying irregular mucosa in the posterior anal canal (**E**).

Nevertheless, interpreting DWI remains complex due to the naturally high T2 signal of mucin (as illustrated in Figure 5). This is compounded when considering mucin pools distant from the primary tumor site and lymph nodes containing mucin, which further complicate the post-therapy diagnostic process.

#### 4.4.5. Pitfalls

**Figure 6:**
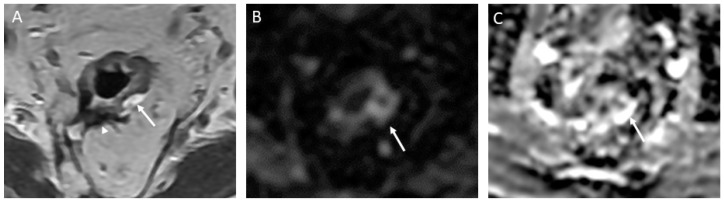
T2 shine-through effect mimicking residual tumor in a 58-year-old man with rectal adenocarcinoma. Axial T2-weighted MR image after the completion of neoadjuvant chemoradiotherapy shows a thick scar along the tumor bed (arrowhead), and mucin extension into the left mesorectal space is noted at the 2:00 to 4:00 position (arrow) (**A**). On the corresponding axial diffusion-weighted image, there is a notable high signal at the same location as the mucin extension (arrow) (**B**). An axial apparent diffusion coefficient map (ADC) further confirms the presence of high signal intensity at the location of the mucin extension (indicated by the arrow) (**C**). This high signal intensity observed on both the diffusion-weighted image and the ADC map indicates the T2 shine-through effect, signifying non-restricted diffusion.

There are a few commonly encountered pitfalls that the radiologist needs to be aware of when evaluating rectal tumor response on MRI. This includes the “T2 shine-through” effect, which refers to the presence of a high signal on DW images without a correlating low signal on the ADC map. This phenomenon is usually the result of a small amount of fluid content in the rectum, which can be mistaken for an incomplete response if interpreted based on the DWI alone. Therefore, any focus of restricted diffusion seen at the tumor bed on DWI should be correlated with low signal change on the ADC map (Figure 6). Additionally, studies have reported that the configuration of the signal on DWI should be considered; trace fluid will have a more trident appearance than the mass-like shape of residual tumor. It is important to note that mucinous tumors commonly show T2 shine-through due to their high mucinous content.

**Figure 7:**
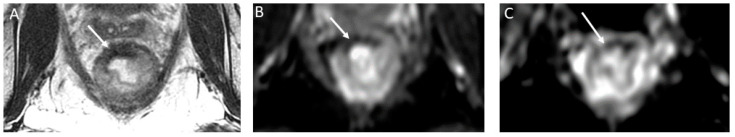
T2 dark-through effect and rectal contents causing restricted diffusion and mimicking residual tumor in a 54-year-old man with rectal adenocarcinoma. (**A**) Axial T2-weighted MR image after the completion of neoadjuvant chemoradiotherapy shows a thin hypointense scar at the tumor bed (arrow). (**B**) Axial diffusion-weighted image shows low signal intensity within the tumor bed (arrow). (**C**) Corresponding axial apparent diffusion coefficient (ADC) map shows low signal intensity at this site (arrow). T2 dark-through shows the rectal wall scar with low signal intensity on T2-weighted imaging, diffusion-weighted imaging, and the ADC map due to fibrosis. Viable tumor should be considered only in cases with high signal intensity on diffusion-weighted imaging and low signal intensity on the ADC map within the tumor bed. Comparison with baseline scans may also be of value.

On the other hand, “T2 dark-through” is when extensive post-treatment fibrosis results in a low signal on the ADC map. This phenomenon results in part from the high collagen content of fibrosis. To avoid this, the reader should correlate the low signal seen on the ADC map with the signal on DWI and T2-weighted imaging; if the signal is low on all sequences, then it should be interpreted as fibrosis (Figure 7) [73].

**Figure 8:**
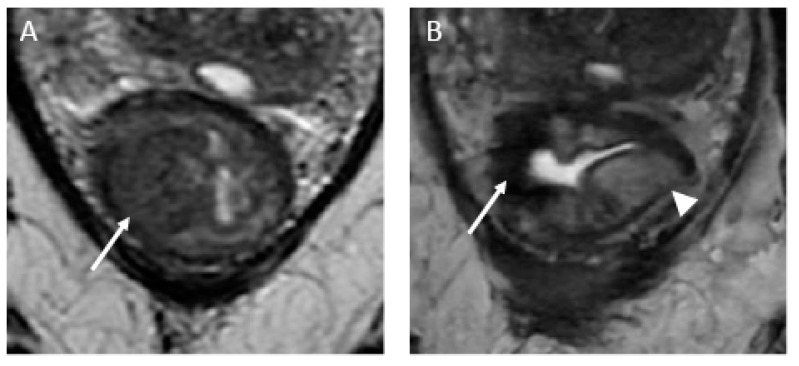
Submucosal edema mimicking residual tumor in the uninvolved rectal wall in a 47-year-old man with rectal adenocarcinoma. (**A**) Baseline axial T2-weighted image shows a semicircumferential rectal tumor extending between the 7 o’clock and 12 o’clock positions (arrow). (**B**) Axial T2-weighted image after the completion of neoadjuvant chemoradiotherapy shows a complete response appearance, characterized by a crescent-shaped scar within the mucosal/submucosal layers (arrow). Note the high signal intensity at the uninvolved rectal wall (arrowhead), representing submucosal edema. Response assessment should not be based on the appearance of uninvolved parts of the rectum.

As mentioned previously, post-treatment changes include submucosal edema at the tumor bed, particularly on the rectal wall opposite the treated mass. Submucosal edema will result in wall thickening with high-to-intermediate signal on T2-weighted imaging, which can be mistaken as a rectal mass, commonly referred to as a pseudotumor in these cases (Figure 8). To avoid this, it is crucial to evaluate the baseline (pre-treatment) rectal MRI when available [76].

### 4.5. How to Evaluate Mesorectal Fascia Status

The most crucial element of all in the treatment assessment of rectal cancer is the reassessment of the mesorectal fascia (MRF). Achieving clearance of the MRF on restaging high-resolution T2-weighted MRI holds a positive predictive value of up to 90% for a clear margin upon pathological examination, which may support a shift toward less invasive surgical approaches [77,78]. Conversely, when the MRF is approached by dense hypointense fibrosis or by an intermediate tumor signal intensity, the MRF should be considered involved for both, even though the likelihood of tumor presence upon pathological assessment is lower for the former as compared to the latter, since distinguishing purely fibrotic tissue from fibrosis with residual tumor cells is challenging [77,78].

The updated lexicon by the SAR DFP employs the term “MRF,” which is an anatomical term, as opposed to circumferential resection margin (CRM), which relates to the operative surgical margin, dependent on the surgical approach [63].

The status of the MRF hinges on the shortest distance between the MRF and the outermost part of the rectal tumor, including extramural vascular invasion, tumor deposits, or disrupted capsule-positive lymph nodes [79]. Lymph nodes with intact capsules are not considered involved, as they do not correlate with increased local recurrence rates [79,80]. The SAR DFP template utilizes a three-tiered system for MRF status: “involved” for a distance less than 0.1 cm, “threatened” for a distance of 0.1–0.2 cm, and “clear” for a distance of more than 0.2 cm [81].

DWI may also play a role in predicting MRF status [82]. However, it often overestimates the disease extent, particularly in anterior locations and in tumors close to the anal verge, as confirmed by a whole-mount study [83].

### 4.6. How to Evaluate Rxtrarectal Disease

CR, nCR, and iCR responses are also contingent on the assessment of extrarectal disease. When there are no lymph nodes, EMVI, or tumor deposits, we categorize the response as CR. In cases where any of these factors fall into borderline territory, the response is categorized as nCR. However, if any of these factors raise suspicion, the response is deemed iCR.

#### 4.6.1. Lymph Nodes

When evaluating the lymph nodes, it is important to note that after neoadjuvant therapy, most lymph nodes typically shrink or even vanish on MRI [84]. MRI becomes more accurate after neoadjuvant therapy compared to the initial assessment, with the capability of identifying patients without any signs of cancer in their lymph nodes with up to 95% accuracy [85].

As this review will clarify, unlike the baseline assessment that utilizes morphological criteria for the assessment of lymph nodes, at restaging, the focus is solely on size.

There are different types of lymph nodes in the pelvic area. Some are close to the rectum (mesorectal, obturator, and internal iliac nodes) and are considered “locoregional” nodes (N+ disease involves only these nodes), while others are farther away (common iliac nodes, external iliac nodes, and inguinal nodes) and are considered “non-regional” nodes (M1 disease involves these nodes and suggests that the disease has spread). There is an exception: if a lower rectal tumor goes below the dentate line (not visible on MRI but roughly in the middle of the anal canal), then the inguinal lymph nodes become “locoregional” again [86,87].

An additional update concerns N classification, as the SAR DFP recommends using “N+” to indicate abnormal locoregional lymph nodes or tumor deposits on MRI and “N−” to indicate the absence of locoregional nodal disease instead of specifying N0, N1a, N1b, N1c, or N2 categories [81].

#### 4.6.2. Mesorectal Lymph Nodes

When assessing mesorectal lymph nodes, the most consistent finding on MRI that is associated with negative mesorectal lymph node status upon pathological examination is the absence of lymph node visualization on DWI [88]. However, in general, mesorectal lymph nodes measuring less than 0.5 cm in the short axis should be considered indicative of a negative status [89,90] despite the existence of false-positive and false-negative cases.

#### 4.6.3. Lateral Pelvic Lymph Nodes

For lateral pelvic lymph nodes, morphologic criteria have not been shown to be helpful at baseline or restaging MRI. On the other hand, according to recommendations from the Lateral Node Study Consortium [90], during restaging, the persistence of an internal iliac lymph node measuring > 0.4 cm or an obturator lymph node measuring > 0.6 cm after neoadjuvant CRT is regarded as suspicious, given their association with local and distant recurrence [91]. Based on size criteria, when the lateral pelvic lymph nodes are positive on imaging, a boost of radiotherapy and/or a lymph node dissection may be indicated to improve the clinical outcome [91].

#### 4.6.4. Non-Locoregional/Distant Lymph Nodes

Lymph nodes outside the regional area may raise suspicion if they measure more than 1.0 cm in the short axis. However, enlargement of the lymph node’s short axis alone is not a specific indicator of malignancy [37]. It is essential to consider factors such as the tumor’s location, expected drainage pattern, and malignant features like abnormal signals in the parenchyma, irregular node borders, asymmetry, and spherical shape.

#### 4.6.5. Tumor Deposit/ EMVI

A tumor deposit is described as a tumoral lesion located within the mesorectum, disconnected from the primary tumor, and devoid of lymphoid tissue upon pathological examination [92]. On imaging, distinguishing a tumor deposit from a lymph node can be challenging; typically, tumor deposits manifest as irregular nodules closely associated with blood vessels, making separation difficult. When a tumor deposit is identified, it falls under the classification of N1c disease according to the 8th edition of the American Joint Committee on Cancer classification. The presence of a tumor deposit often coexists with EMVI and is linked to a worse oncological prognosis, with a high incidence of liver and lung metastasis [93].

On MRI, EMVI is typically identified by prominent tubular structures exhibiting intermediate signal intensity on T2-weighted imaging and diffusion restriction within the vessel, which originate from the tumor bed, with or without irregular contours. Regression of EMVI following neoadjuvant therapy is linked to enhanced survival compared to persistent EMVI [94]; however, the presence of edema, desmoplastic reaction, and inflammatory changes can limit the effectiveness of MRI in identifying viable tumors within EMVI or tumor deposits. DWI serves as an additional tool to enhance EMVI detection [95].

A recent study showed that DWI has high specificity and moderate sensitivity for the detection of viable tumor deposit and EMVI after neoadjuvant therapy, and viable tumor deposit and EMGI detected using DWI is linked with worse oncological prognosis, i.e., a decrease in disease-free survival and overall survival [96].

## 5. Structured Reporting

There are a number of studies that have shown the value of structured reporting in rectal cancer [97,98,99]. Furthermore, American and European societies have proposed lexicons and standardized reports for this purpose [63,81,100].

A recently updated consensus statement from the SAR proposes the following terminology for treatment response assessment in rectal cancer [63]:

- CR and nCR response categories are grouped together because they can be closely monitored safely, and most cases of nCR will reach CR at 6–12 weeks after neoadjuvant therapy [101]. Both CR and nCR imply that both T2-intermediate signal and restricted diffusion have resolved entirely or almost completely.
- iCR should be applied when, even though the tumor volume has decreased, there is residual T2-intermediate signal and/or restricted diffusion.
- The term recurrence should be used only after local excision or total mesorectal excision, while the term regrowth should be used after chemotherapy or RT. The latter applies when, after having documented CR, there is a new tumor in the bowel wall (local), adjacent structures (loco-regional), or lymph nodes. Dowel wall regrowth can be suspected when a prior low-signal intensity scar is a new area of T2-intermediate signal or restricted diffusion, thickening, or heterogeneity [63].

Also, on the SAR webpage, a structured reporting template to evaluate the response to treatment is available [102]. This structured reporting template lists the MRI features to evaluate in different scenarios. These features include the following:

- Restricted diffusion and low ADC in the tumor or tumor bed: present, absent, or artifact/equivocal/not available.
- T2 signal intensity in the tumor or tumor bed: intermediate, mixed, entirely dark, nearly normalized appearance of rectal wall, or bright mucin.
- Distance of the inferior margin of the treated tumor to the anal verge and to the top of the sphincter complex/anorectal junction.
- Relationship of the treated tumor to the anterior peritoneal reflection: above, straddles, or below.
- Craniocaudal length and maximal wall thickness (current and pre-treatment measurements for both features).
- EMVI: no (no EMVI evident at pre-treatment imaging), no (complete regression), yes (partial regression), or yes (unchanged from baseline).
- Shortest distance of tumor/fibrosis to the MRF.
- Tumor deposit, lymph node, or EMVI threatening the MRF: yes or no.
- In the case of a low-rectal tumor, is there an invasion of the anal sphincter complex? no, extends into the internal sphincter, extends into intersphincteric space, or extends into or through the external sphincter.
- Lymph nodes and/or tumor deposits: mesorectal/superior rectal or extra-mesorectal.

## 6. Future Directions

### 6.1. Fluorodeoxyglucose Positron Emission Tomography (FDG PET)

FDG-PET is a functional imaging modality utilized to visualize and assess metabolic activity within the body’s tissues. Previous reports have established its utility, particularly in the realm of oncologic clinical practice and especially in evaluating treatment response. Nonetheless, the application of FDG PET in rectal cancer has lagged behind that of other cancers due to the impact of peristaltic movements in the rectum that have a detrimental effect on the quality of FDG PET images. Recent advances in hybrid PET/computed tomography (CT) technology have, however, mitigated these issues, enabling higher spatial resolutions and reduced imaging artifacts. In a recent meta-analysis by Lee et al., comprising 9 studies involving 427 patients, ^18^F-FDG PET/CT demonstrated comparable diagnostic performance to MRI in predicting pathologic response following neoadjuvant CRT in rectal cancer patients [103]. Furthermore, a report by Ince et al. highlighted the potential of FDG PET/MRI, which achieved a remarkable 100% accuracy in assessing pathological complete response. This accuracy is attributed to the ability of FDG PET/MRI to detect residual disease that may be missed by MRI alone [104]. A PET/MRI scanner, as a state-of-the-art medical imaging device, seamlessly integrates two potent imaging modalities: PET and MRI. This hybrid system facilitates the concurrent acquisition of functional and anatomical data within a single scan, offering the potential for more precise diagnostic assessments of treatment responses in patients with rectal cancer.

### 6.2. Radiomics and Personalized Medicine

Advancements in the diagnosis and treatment of rectal cancer, particularly through the implementation of total mesorectal excision and neoadjuvant therapy, have led to a notable reduction in local recurrence rates. With the increasing intensity and broader utilization of neoadjuvant therapy for the purpose of enhancing organ preservation rates, especially in patients with early tumor stages, the accurate evaluation of treatment response is crucial [105,106,107]. In the context of future personalized medicine, the ability to predict treatment response prior to the initiation of treatment is of paramount importance to identify which patients are most likely to benefit from intensified neoadjuvant therapy and to prevent unnecessary toxicity from intensified neoadjuvant therapy in patients who are not likely to benefit from this but who will require total mesorectal excision instead.

Radiomics represents a state-of-the-art approach involving the extraction and comprehensive analysis of numerous quantitative features from medical images. These features encompass a broad spectrum of information, encompassing aspects such as size, shape, texture, intensity, and spatial relationships within the image structures. Radiomics primarily transforms routine medical imaging modalities, including CT, MRI, and PET scans, into high-dimensional datasets amenable to advanced machine learning techniques. A recent meta-analysis conducted by Tanaka et al., which incorporated findings from 16 studies, revealed that radiomic models designed for the prediction of response to neoadjuvant therapy in patients with rectal cancer demonstrate promising predictive potential. Of note, two models based on collagen features exhibited the most robust predictive performance, with areas under the curve ranging from 0.83 to 0.91 and favorable calibration [108]. However, given the potential for bias in these studies, the utility of previously reported radiomic models should be validated in the future through well-designed, prospective studies involving large population cohorts.

## 7. Conclusions

In conclusion, this review comprehensively examines the advancements and efficacy of MRI in evaluating rectal cancer following neoadjuvant therapy. The findings underscore MRI’s critical role in treatment planning and outcome prediction, highlighting its superior accuracy and detail compared to other imaging modalities. However, challenges such as variability in interpretation and technological limitations persist, necessitating further standardization and innovation. Future research should focus on integrating advanced MRI techniques with emerging treatment approaches to optimize patient outcomes. The integration of MRI in rectal cancer management exemplifies a significant stride in personalized medicine, offering a promising direction for enhancing patient care.
